# The ERα coactivator, HER4/4ICD, regulates progesterone receptor expression in normal and malignant breast epithelium

**DOI:** 10.1186/1476-4598-9-150

**Published:** 2010-06-15

**Authors:** Jerzy Rokicki, Partha M Das, Jennifer M Giltnane, Olivia Wansbury, David L Rimm, Beatrice A Howard, Frank E Jones

**Affiliations:** 1 Department of Cell and Molecular Biology, Tulane University, New Orleans, Louisiana, 70118, USA; 2 Department of Pathology, Yale University School of Medicine, New Haven, Connecticut, 06520, USA; 3 The Breakthrough Breast Cancer Research Centre, The Institute of Cancer Research, London, SW36JB, UK

## Abstract

The HER4 intracellular domain (4ICD) is a potent estrogen receptor (ERα) coactivator with activities in breast cancer and the developing mammary gland that appear to overlap with progesterone receptor (PgR). In fact, 4ICD has recently emerged as an important regulator and predictor of tamoxifen response, a role previously thought to be fulfilled by PgR. Here we investigated the possibility that the 4ICD coactivator regulates PgR expression thereby providing a mechanistic explanation for their partially overlapping activities in breast cancer. We show that 4ICD is both sufficient and necessary to potentiate estrogen stimulation of gene expression. Suppression of HER4/4ICD expression in the MCF-7 breast tumor cell line completely eliminated estrogen stimulated expression of PgR. In addition, the HER4/4ICD negative MCF-7 variant, TamR, failed to express PgR in response to estrogen. Reintroduction of wild-type HER4 but not the γ-secretase processing mutant HER4V673I into the TamR cell line restored PgR expression indicating that 4ICD is an essential PgR coactivator in breast tumor cells. These results were substantiated *in vivo *using two different physiologically relevant experimental systems. In the mouse mammary gland estrogen regulates expression of PgR-A whereas expression of PgR-B is estrogen independent. Consistent with a role for 4ICD in estrogen regulated PgR expression *in vivo*, PgR-A, but not PgR-B, expression was abolished in HER4-null mouse mammary glands during pregnancy. Coexpression of PgR and 4ICD is also commonly observed in ERα positive breast carcinomas. Using quantitative AQUA IHC technology we found that 4ICD potentiated PgR expression in primary breast tumors and the highest levels of PgR expression required coexpression of ERα and the 4ICD coactivator. In summary, our results provide compelling evidence that 4ICD is a physiologically important ERα coactivator and 4ICD cooperates with ERα to potentiate PgR expression in the normal and malignant breast. We propose that direct coupling of these signaling pathways may have important implications for mammary development, breast carcinogenesis, and patient response to endocrine therapy.

## Findings

Breast cancer is the most frequently diagnosed cancer in North American women and the second leading cause of cancer related death. The natural history, as well as the clinical outcome, of breast cancer are dependent upon the interplay between multiple growth promoting pathways with the estrogen receptor α (ERα) among the most important. In fact, ERα is expressed in up to 75% of primary breast tumors [1] and therapeutic management of patients with ERα positive tumors will involve an endocrine component with tamoxifen being the most commonly prescribed. The high percentage of breast tumors with *de novo *or acquired resistance to tamoxifen has prompted clinicians and researchers to investigate additional predictive markers for endocrine therapy response. One reasonable tumor marker is the estrogen regulated progesterone receptor (PgR). The prevailing paradigm suggests that lack of PgR tumor expression indicates disengaged ERα signaling and therefore concomitant loss of the ERα tumor growth signal and therapeutic target. However, preclinical studies demonstrate that ERα signaling remains intact in tamoxifen resistant cells despite the loss of PgR expression [2].

In a recent study, loss of tumor expression of the receptor tyrosine kinase HER4/ERBB4 (referred to here as HER4) was an independent marker for tamoxifen resistance [3]. HER4 has emerged as a unique cell-surface receptor and several novel HER4 signaling activities in breast cancer are in fact mediated by an independently signaling HER4 intracellular domain (4ICD). For example, 4ICD is a potent ERα coactivator selectively regulating gene expression and breast tumor cell proliferation in response to estrogen [4,5]. In addition, our group has recently demonstrated that tamoxifen disruption of the 4ICD/ERα transcriptional complex results in breast tumor cell killing, in part, by driving mitochondrial accumulation of 4ICD [6]. Within the mitochondria, 4ICD functions as a proapoptotic BH3-only protein [4,6-8]. Accordingly, cytosolic 4ICD is associated with tumor cell apoptosis and improved patient prognosis [9]. The role of 4ICD as an ERα coactivator and the apparent overlapping ability of 4ICD and PgR to predict patient outcome to tamoxifen raises the possibility that 4ICD regulates estrogen stimulated PgR expression in the breast. However, the ERα coactivator function of 4ICD remains to be validated *in vivo*.

**Figure 1:**
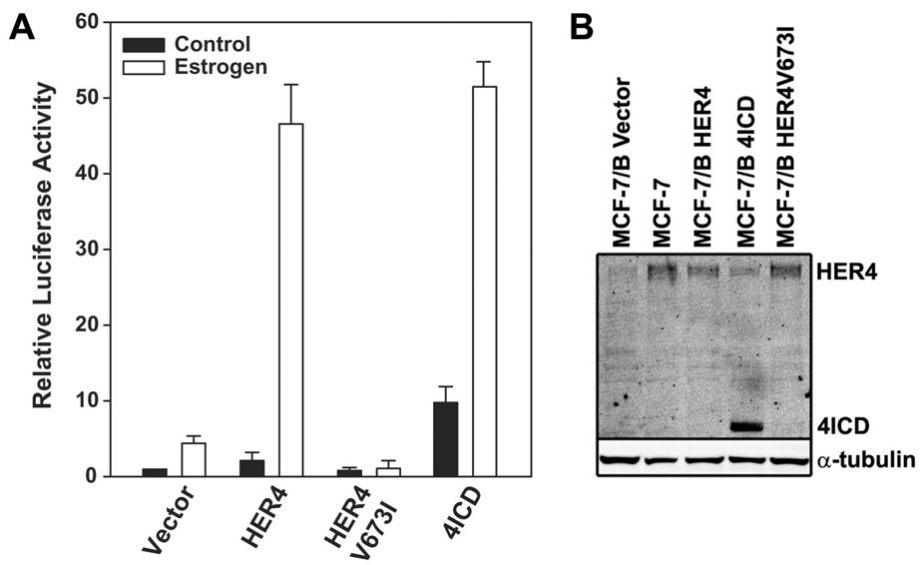
**The HER4 intracellular domain (4ICD) is necessary and sufficient to potentiate estrogen stimulated gene expression**. (A) MCF-7/B (MCF-7 cells with ectopic BCL-2 expression) [22] breast cancer cells were incubated in growth media supplemented with 5% charcoal-stripped FBS (CS-FBS) for 24 hrs and cotransfected with 200 ng of ERE-luc and the indicated HER4 (JM-a, Cyt1 isoform) or 4ICD expression vectors. At 24 hrs post-transfection cells were cultured in the presence or absence of 100 pM 17-β-estradiol (Estrogen) and the luciferase reporter gene assay was performed after 16 hrs as described previously [5]. Each sample was prepared in duplicate and the entire experiment was repeated at least three times. Data represents the mean and standard error of luciferase activity normalized to untreated cells transfected with ERE-Luc alone. (B) Protein lysates prepared from the same experimental treatments were analyzed by western blot for HER4/4ICD (Cell Signaling 111B2) expression with β-tubulin (Upstate DM1A) included as a loading control.

We and others have shown that HER4 potentiates estrogen stimulated expression of an ERE-luciferase reporter [5,10]. We further demonstrated that inactivation of the γ-secretase processing site within HER4 (HER4V673I) prevents generation of 4ICD and abolished HER4/4ICD coactivator activity [8]. We next wanted to determine if independently expressed 4ICD was sufficient to coactivate ERα and enhance estrogen stimulated gene expression. HER4 potentiated estrogen stimulated expression of an ERE-luciferase reporter by over 10 fold whereas the γ-secretase processing mutant, HER4V673I, failed to impact estrogen stimulated gene expression (Figure 1A) [5]. Western blot analysis of transfected cell lysates indicated equivalent levels of HER4 and HER4V673I expression (Figure 1B). Similar to HER4, independently expressed 4ICD enhanced estrogen stimulated gene expression by over 10-fold (Figure 1A). Taken together these results indicate that 4ICD is both necessary and sufficient to coactivate estrogen stimulated gene expression.

**Figure 2:**
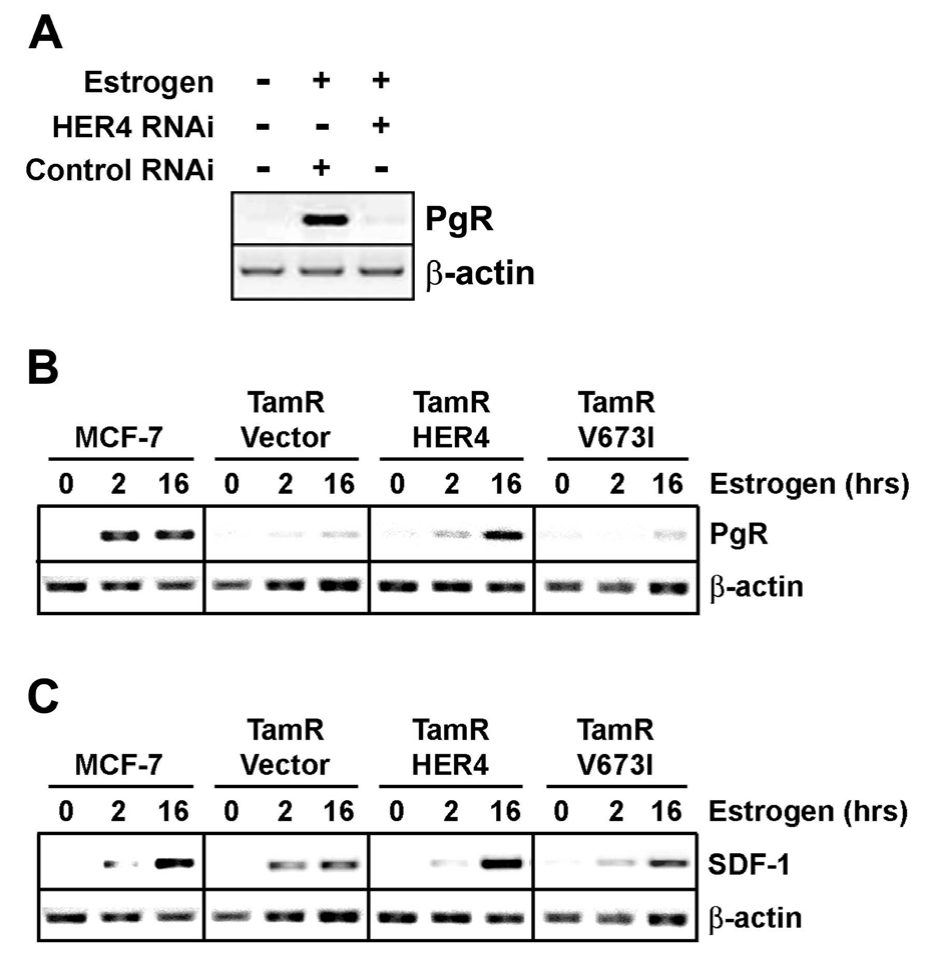
**4ICD is required for estrogen stimulated expression of PgR**. (A) Suppression of endogenous HER4 expression was performed in MCF-7 cells using erbB-4/Her4 siRNA SMARTpool or Nonspecific siRNA Negative Control Pool and siIMPORTER transfection reagent (Upstate Biotechnology, Lake Placid, NY) as described elsewhere [5]. Transfected cells were cultured in growth media supplemented with 5% CS-FBS for 48 hrs and incubated in the presence or absence of 100 pM 17-β-estradiol (Estrogen) for an additional 16 hrs. Total RNA was extracted and PgR expression was analyzed by RT-PCR. β-actin RNA was amplified as a control for RNA quantitation. (B) The MCF-7 variant TamR [23] lacking endogenous HER4 expression [6] was stably transfected with an EGFP vector, HER4-EGFP, or the γ-secretase processing mutant HER4V673I-EGFP which fails to generate 4ICD. Each cell line was incubated in growth media supplemented with 5% CS-FBS for 48 hrs and incubated in the presence or absence of 100 pM 17-β-estradiol (Estrogen) for 2 or 16 hrs. Total RNA was extracted and (B) PgR or (C) SDF-1 expression was analyzed by RT-PCR. β-actin RNA was amplified as a control for RNA quantitation. All RT-PCR reactions and conditions including primer sequences have been described elsewhere [5]. Results indicate that the 4ICD coactivator is essential for estrogen stimulated PgR and SDF-1 expression in the MCF-7 variant.

We next determined the impact of HER4 expression on estrogen stimulated expression of endogenous PgR in the ERα-positive MCF-7 breast tumor cell line. RNAi mediated knockdown of endogenous HER4 completely abolished the ability of estrogen to stimulate expression of PgR in MCF-7 cells (Figure 2A). We substantiated these results in the MCF-7 variant TamR which lacks endogenous HER4 expression [6]. Estrogen stimulation of wild-type MCF-7 cells significantly upregulated PgR expression, however, estrogen failed to stimulate significant levels of PgR expression in the TamR cells (Figure 2B). Reintroduction of HER4, but not the γ-secretase processing mutant HER4V673I, restored estrogen regulation of PgR expression (Figure 2B). Therefore, the ability of HER4 to restore endogenous PgR expression requires γ-secretase processing to generate the 4ICD coactivator. Low expression levels of another 4ICD coregulated gene, stromal cell-derived factor 1 (SDF-1) [5], was observed in the TamR and HER4V673I cell lines, but similar to PgR optimal SDF-1 expression required reintroduction of HER4 (Figure 2C).

**Figure 3:**
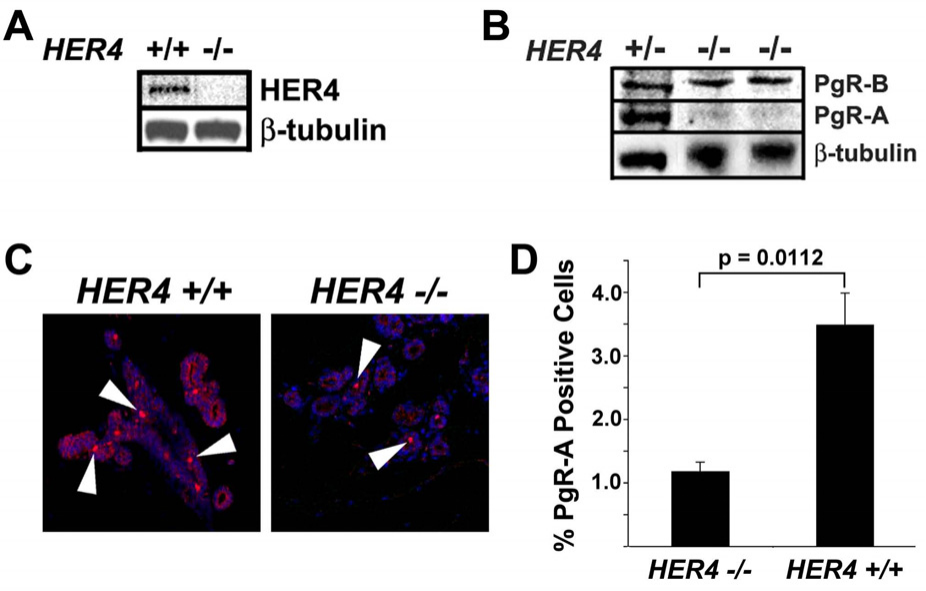
**HER4 regulates PgR expression in the mouse mammary gland**. (A) Transgenic mice expressing HER4 under the control of the cardiac specific myosin promoter were crossed with *HER4+/- *mice (both kindly provided by Jon Golding, Open University) to generate *HER4-/-*;HER4^heart ^(*HER4-/-*) mice [17]. Mammary glands were collected and snap frozen in liquid nitrogen or spread on a glass slide, fixed overnight in formalin, processed, and paraffin embedded. Lysates were prepared from P14.5 mammary glands and analyzed by western blot using an antibody that recognizes (A) HER4 (Cell Signaling 111B2) or (B) both PgR-A and PgR-B isoforms (DAKO A0098). (C) Immunofluorescent localization of PgR-A in mammary glands at P14.5. After antigen retrieval by pressure cooker treatment in citrate buffer, sections were incubated in peroxidase block followed by DAKO block before overnight incubation with the PgR-A specific anti-PgR sc-538 (Santa Cruz) [18] antibody. Secondary antibody was Alexa 555-conjugated (Invitrogen) and nuclei were counterstained with DAPI. Arrowheads indicate nuclear staining of PgR-A. (D) The number of PgR-A-positive alveolar cells were quantitated from images captured using a TCS SP2 Leica confocal microscope with an Acousto-Optical Beam Splitter. Mammary glands from three mice per genotype were analyzed and a minimum of 1000 alveolar cells per mouse were counted. Results are expressed as mean +/-SEM. Statistical significance was determined using the Student's *t *test. A significant (p = 0.0112) 2.95-fold reduction in PgR-A positive alveolar cells was observed in mammary glands lacking HER4 expression when compared to wild-type control mammary glands.

We next examined the developing mouse mammary gland to confirm 4ICD coactivation of PgR expression *in vivo*. There is significant phenotypic overlap between HER4, ERα, and PgR deficient mammary glands. Specifically, each receptor appears to be essential for pregnancy-induced lobuloalveolar development [11-17]. Furthermore, we have observed dramatic nuclear localization of the 4ICD coactivator within mammary epithelium coincident with the essential role of HER4 during pregnancy [4,13]. To determine if 4ICD coactivates PgR expression *in vivo *we examined expression of the PgR isoforms PgR-A and PgR-B in *HER4*-null mammary glands by western blot (Figure 3A). Significantly, at 14.5 days post-coitus (P14.5) PgR-A expression was suppressed in the HER4 deficient mammary glands whereas PgR-B expression appeared to be unaffected (Figure 3B). This result was confirmed by immunohistochemistry (IHC) where PgR-A expression was significantly suppressed in HER4 deficient mammary glands at P14.5 (Figure 3C and 3D). Interestingly, PgR-A, but not PgR-B, is estrogen regulated and coexpressed with ERα in the mammary gland [18]. These observations and our results strongly implicate the 4ICD/ERα transcriptional complex as an important regulator of *in vivo *PgR-A expression in the mammary gland.

**Figure 4:**
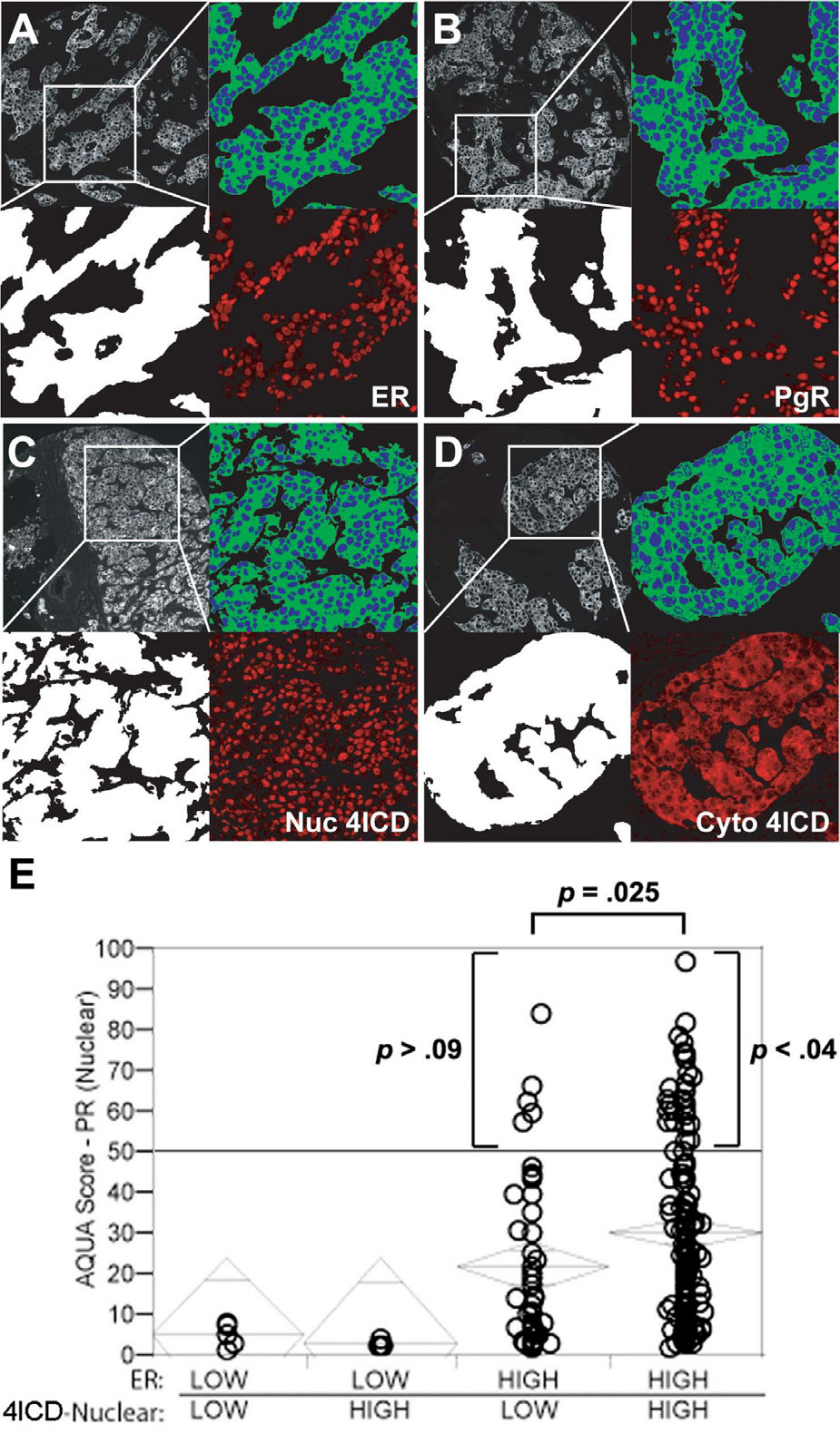
**Nuclear 4ICD coactivates PgR expression in human breast carcinomas**. A cohort of 196 samples of PgR(+) invasive ductal carcinomas from the Yale University Department of Pathology diagnosed from 1961 to 1983 were stained in a tissue microarray format using a modified indirect immunofluoresence method and antibodies described previously [20] with the addition of anti-PgR; PgR636 (Dako) and anti-HER-4 sc-283 (Santa Cruz Biotechnology, Santa Cruz, CA) antibodies. Subcellular immunofluoresence was analyzed by a pathologist using the AQUA™ method as published previously [20,21]. (A-D) Immunofluorescent illustration of AQUA^®^. In each panel, representative pseudo-colored images are shown of cytokeratin (upper left), tumor mask (lower left), nuclear (blue) and non-nuclear or membrane compartments (green) (upper right), and target expression (red) after RESA application (lower right) as described elsewhere [20,21]. (A) ER nuclear expression, (B) PgR nuclear expression, (C) nuclear expression of 4ICD, (D) predominantly cytoplasmic expression of 4ICD. (E) AQUA scores for ERα and nuclear 4ICD were dichotomized to examine the association of these markers with continuous PgR AQUA scores. The cutpoint for ERα was chosen as 3.75 (bottom 25% vs. top 75%) [1]. The cutpoint for nuclear 4ICD was near the cohort median at 10. Chi-square analysis revealed a significant association between PgR and ERα expression (p < .0001). The PgR AQUA score was significantly greater in tumor samples with ERα and nuclear 4ICD coexpression (p = .025). The highest levels of PgR expression (AQUA > 50) were significantly associated with ERα and nuclear 4ICD coexpression (p < .04).

In breast cancer PgR expression represents a clinically important indicator of prognosis. In light of recent evidence indicating that the 4ICD coactivator is an important effector of tumor response to tamoxifen [6] and possibly fulvestrant [19], 4ICD coregulation of PgR expression in breast tumors could have important clinical implications. We used a sophisticated automated quantitative analysis (AQUA) algorithm [20,21] to measure levels of ER, PgR, and nuclear or cytoplasmic 4ICD in primary human breast tumors by IHC (Figure 4A-D). AQUA analysis allows detection of continuous and subtle differences in tumor protein levels by IHC. To determine if 4ICD coactivates PgR expression in breast carcinomas we quantitated the levels of PgR expression in 196 therapy naive PgR positive breast tumors using the AQUA IHC procedure [20,21].

Consistent with other clinical studies, chi-square analysis of patient groups selected by ERα expression demonstrated a lack of PgR expression in the absence of ERα (Figure 4E) (p < 0.0001). Although ERα expression alone was sufficient to promote PgR expression in breast tumors, the levels of PgR expression were significantly potentiated in tumors coexpressing ERα with nuclear 4ICD (PgR AQUA 21.7 vs. 30.0; p = 0.025) indicating that the 4ICD coactivator potentiates PgR expression in breast tumors. This association was significantly strengthened at the highest levels of PgR expression. In fact, PgR AQUA levels > 50 were significantly associated with ERα and nuclear 4ICD coexpression (p < 0.04) but not ERα expression alone (p > 0.09) (Figure 4E). These observations demonstrate that the 4ICD coactivator potentiates ERα regulated PgR expression during breast tumorigenesis and robust tumor expression of PgR requires ERα coexpression with the 4ICD coactivator. These results further implicate 4ICD as an important ERα coactivator regulating estrogen stimulated gene expression in primary breast tumors.

## Conclusions

In summary, we provide compelling evidence from multiple *in vitro *and *in vivo *experimental systems that the 4ICD transcriptional coactivator cooperates with ERα to regulate PgR expression in the normal and malignant breast. The potential clinical impact of these findings is underscored by the recent emergence of 4ICD as a critical effector of tamoxifen action [6] as well as an important tumor marker predicting patient response to tamoxifen [3,6]. Our results suggest that PgR expression represents a clinical surrogate marker for tumor expression of 4ICD, a potent ERα coactivator and regulator of tamoxifen action.

## List of Abbreviations

4ICD: HER4 intracellular domain; AQUA: automated quantitative analysis; BH3: BCL-2 homology domain 3; ERα: estrogen receptor alpha; IHC: immunohistochemistry; PgR: progesterone receptor; PgR-A: progesterone receptor isoform A; PgR-B: progesterone receptor isoform B; SDF-1: stromal cell-derived factor 1

## Competing interests

The authors declare that they have no competing interests.

## Authors' contributions

JR performed all expression assays in cell lines and assisted in manuscript preparation. PMD performed HER4/4ICD western blots and prepared the revised manuscript. JMG performed AQUA IHC of primary breast tumors. OW performed expression and IHC analyses in mouse mammary glands. DLR designed and provided statistical analysis of AQUA data. BAH designed and analyzed data from the mouse mammary gland expression studies. FEJ conceived of the study, participated in data analysis at each stage, and wrote the manuscript. All authors read and approved the final publication.
